# Is it about substituting an addiction with another? development and initial psychometric properties of the first heated tobacco products addiction questionnaire (HeaTPAQ)

**DOI:** 10.1186/s13722-025-00551-4

**Published:** 2025-02-26

**Authors:** Feten Fekih-Romdhane, Rabih Hallit, Diana Malaeb, Fouad Sakr, Mariam Dabbous, Sahar Obeid, Souheil Hallit

**Affiliations:** 1 The Tunisian Center of Early Intervention in Psychosis, Department of Psychiatry “Ibn Omrane”, Razi hospital, Manouba, 2010 Tunisia; 2 Faculty of Medicine of Tunis, Tunis El Manar University, Tunis, Tunisia; 3 School of Medicine and Medical Sciences, Holy Spirit University of Kaslik, P.O. Box 446, Jounieh, Lebanon; 4 Department of Infectious Disease, Notre Dame Secours University Hospital Center, Street 93, Byblos, Postal Code 3, Byblos, Lebanon; 5 Department of Infectious Disease, Bellevue Medical Center, Mansourieh, Lebanon; 6 College of Pharmacy, Gulf Medical University, Ajman, United Arab Emirates; 7 School of Pharmacy, Lebanese International University, Beirut, Lebanon; 8 Social and Education Sciences Department, School of Arts and Sciences, Lebanese American University, Jbeil, Lebanon; 9 Psychology Department, College of Humanities, Effat University, Jeddah, 21478 Saudi Arabia; 10 Applied Science Research Center, Applied Science Private University, Amman, Jordan

**Keywords:** Heated tobacco products, Addiction, Nicotine, Scale development, Scale validation, Psychometric properties

## Abstract

**Background:**

Public health experts currently agree that heated tobacco products (HTPs) pose a significant health risk for their consumers. The same concentrations and speed of delivery of nicotine found for HTPs and conventional combustion cigarettes make it necessary to consider the addictiveness of HTPs, and provide precise diagnostic instruments to serve as the basis for effective treatment plans. Therefore, the main objectives of this study were to design a questionnaire for HTPs addiction called “*Hea*ted *T*obacco *P*roducts *A*ddiction *Q*uestionnaire (HeaTPAQ)” and to examine its psychometric properties.

**Methods:**

Adults from the general population of Lebanon (*n* = 754) were administered the HeatPAQ, along with the Fagerström test for nicotine dependence (FTND), the Caffeine Use Disorder Questionnaire, the Generalized Anxiety Disorder 7-item, and the Patient Health Questionnaire-9. We split the main sample into two subsamples; subsample 1 consisting of 33% of the participants used for the exploratory factor analysis (EFA) (*n* = 246; mean age 27.82 ± 9.38 years) and subsample 2 consisting of 67% of the participants used for the confirmatory factor analysis (CFA) (*n* = 508; mean age 27.81 ± 8.80 years).

**Results:**

EFA then CFA analyses revealed a one-factor model consisting of 13 items with acceptable fit to the data. The HeaTPAQ reached excellent internal consistency coefficients, with both Cronbach’s α and McDonald’s ω values of 0.96. The one-dimensional structure of the HeaTPAQ was found to be invariant across sex groups. Convergent validity was demonstrated through significant positive correlation with FTND scores. Furthermore, HeaTPAQ scores correlated positively with measures of caffeine addiction, anxiety and depression, which suggests the adequate concurrent validity of the scale.

**Conclusion:**

Findings suggest that the HeatPAQ is a specific, short and simple-to-use self-report questionnaire to assess HTPs addiction reliably and validly. Pending future studies confirming our results, we hope that the HeatPAQ will facilitate routine screening for HTPs addiction, which is an essential step towards appropriate prevention and intervention efforts and to inform policy makers.

**Supplementary Information:**

The online version contains supplementary material available at 10.1186/s13722-025-00551-4.

## Introduction

According to the World Health Organization (WHO), tobacco usage is one of the leading causes of premature death and one of the biggest public health threats worldwide [1]. As an alternative to traditional cigarettes, novel nicotine delivery devices or heated tobacco products (HTPs) were introduced to the international market. In 2014, a new type of HTP named IQOS (“I-Quit-Ordinary-Smoking”) from Philip Morris International (PMI) was introduced to the market [2–4], and has become the most popular and widely available HTP product worldwide [5]. Other brands also exist, such as Ploom (Japan Tobacco International), Pax (Pax lab) and Glo (British American Tobacco). Over the recent years, HTPs have experienced a rapid surge in sales and have become increasingly popular, with a reported 2,000% increase from 2018 to 2020 in the European union [6]. HTPs have been made available in tens of countries and all regions of the world [7]. Young people may be particularly encouraged to use heated tobacco smoking technology, especially because of the introduction of attractive tastes (e.g., sweet fruit) of tobacco sticks. Other commonly endorsed motives for HTPs use include curiosity and novelty-seeking, ease of use/convenience, affect regulation (stress and boredom relief), and the perception that they are healthier alternatives to waterpipes and cigarettes [8]. Although it is widely admitted that HTPs produce fewer and lower levels of toxic chemicals than conventional cigarette smoke, they are still not free of risks. Indeed, a key consideration to bear in mind when interpreting previous findings is that more than half of the studies on HTPs exposure and health impacts has been sponsored and provided by the tobacco industry [9]. The body of knowledge coming from studies posing a potential conflict of interest did not allow the recognition of HTPs as being “reduced risk products” by leading health organizations (e.g [10–12]).,.

### Health risks associated with HTPs use

In January 2018, the Food and Drug Administration (FDA) Tobacco Products Scientific Advisory Committee stated that no clear scientific evidence exists to support that IQOS use is less harmful than continuing conventional cigarette use or that it could eliminate the risk of tobacco-related diseases [13, 14]. HTPs contain some toxicants that are not present [15] or present at lower amounts [16] than in combustible cigarettes. Evidence from independent human-based research not sponsored by the tobacco industry suggests that harmful constituents and toxic chemicals are not totally removed from the HTP aerosol, and that active and passive HTP smoking might have potentially detrimental effects on human health (for systematic review, see [9]). The use of HTPs was linked to negative cardiovascular effects similar to those observed with cigarette smoking [17], including increased arterial stiffness and platelet thrombus formation [18], and was found to confer a possible increased risk of unexpected hepatotoxicity not observed during cigarette smoking [19]. In addition, HTPs were shown to have a lower cancer potency than that estimated by traditional cigarette use, but much higher cancer potency compared to most e-cigarettes [20]. Beyond their effects on physical health, HTPs were also found to be highly addictive [21].

### The risk of dependence on HTPs

Most of the limited independent studies focused on the toxicological rather than the addictive effects of HTPs. However, there is sufficient evidence to suggest that HTPs have an addictive potential. Indeed, while IQOS official shop assistants and some official PMI websites inform users that each stick of tobacco contains 0.5 mg of nicotine, the quantity of nicotine actually included in the stick is 8 times as much (4.1 mg) [22]. Several independently funded studies revealed that HTPs contain similar nicotine concentrations in the blood compared to traditional cigarettes [23, 24]. In addition, some HTPs supply nicotine that attains the bloodstream at a delivery speed approaching that reached by inhaling combustible cigarette smoke [25]. Given that the addictive potential of nicotine-delivery systems depends on both the intensity and speed of nicotine delivered to the body [26], it can be assumed that some regular HTPs users might develop both a physical and psychological dependence.

Nevertheless, as previously mentioned, scant research has been carried-out on the addictiveness of HTPs. A Japanese study found that time-to-first HTP use (which is a strong indicator of nicotine dependence) was most frequently within 6–30 min for IQOS users, versus more than 60 min for glo and Ploom TECH users [27]. A Swiss study showed that current IQOS consumers had a medium to high scores of perceived dependence on HTPs, and more than half of them inhaled their first puff within 30 min of waking up [21]. As HTPs use triggers nicotine addiction, it makes IQOS cessation itself difficult. For example, an Italian study showed that 69% of exclusive IQOS users (*N* = 1907) do not intend to quit its use within the next 6 months [28]. Likewise, Queloz and Etter [21] found that 43.6% of IQOS users believed that if they tried to stop using their HTP, the probability of success would be low, and 29.6% thought it would be “very difficult” to “impossible” to definitively stop using the HTP. Some researchers have sounded the alarm about the misleading labelling of IQOS, and the risks inherent to consumers being likely to ‘switch completely’ from smoking cigarettes to using IQOS [14].

Over and above all these physical and mental health risks, there is prospective evidence that using HTPs does not help current smokers quit or former smokers not to relapse, suggesting that “HTPs could serve as a disincentive to successful quitting” [29]. A recent Cochrane Review of randomized controlled trials (RCTs) on the effectiveness and safety of HTPs for smoking cessation revealed that all RCTs were funded by tobacco companies, and that none of them reported smoking cessation outcomes [30]. The European Respiratory Society [11] and other official institutions (e.g [31])., stated that HTPs cannot be recommended for use as a cessation aid. Despite all this evidence, a study indicated that Japanese physicians had low concerns about the addictive potential of HTPs, and that ever-non-HTP smokers reported being significantly more concerned than current HTP smokers (42.7% versus 25.5%) [32]. Moreover, the same study revealed that about a half of the ever-non-HTP smoker physicians (49.1%) asked their patients about using HTPs compared to only 36.1% ever-HTP-smoker physicians [32]. Given the consistent development and steady growth of the HTPs market, as well as the increasing prevalence of its use and magnitude of its impacts on users’ health, there have been urgent calls to conduct more studies independent of commercial interests [12]. This highlights the strong and urgent need to develop a new empirical measure of HTPs addiction, to help address this often-neglected issue in clinical and research contexts.

### Measurement instruments to assess HTPs addiction

Although HTPs are gaining growing attention among addictive substances, there are, to date, no valid instruments for the assessment of HTPs addiction. The assessment of such a potentially addiction through sound psychometric measures is an essential prerequisite for further medical and psychotherapeutic interventions. The existing measures were specifically intended to assess the use of, and dependence to cigarette smoking, such as the Cigarette Dependence Scale (CDS) [33] and the Fagerström Test for Nicotine Dependence (FTND) [34]. PMI designed a measure aimed at assessing global dependence on tobacco and nicotine products, which they called the ABOUT–Dependence (i.e., Assessment of Behavioural OUtcomes related to Tobacco and nicotine products-Dependence) [35, 36]. However, no studies using this tool have been published as far as we are aware of. Sutanto et al. [27] were among the first to measure patterns of HTPs use in community Japanese adults using a single-item question (i.e., “How often, if at all, do you currently use heat-not-burn products? - These include products such as IQOS, Ploom TECH, and glo”). Participants were then classified as current HTPs users if they answered “less than weekly, but at least once a month”, “less than daily, but at least once a week”, and “daily” [27]. To assess perceived dependence on HTPs, other authors resorted to an adaptation and a modification of the Fagerstrom Test, by using a scale from 0 to 100 and replacing the term “cigarette” and “smoking” with “tobacco vaporizer” and “using a tobacco vaporizer”, respectively [21].

The Diagnostic and Statistical Manual of Mental Disorders, Fifth Edition (DSM-5) defines Substance-use disorders as patterns of symptoms resulting from the use and compulsive seeking of a substance despite adverse consequences [37]. The DSM-5 recognizes substance-related disorders resulting from the use of 10 separate classes of drugs, including Tobacco. Although the American Psychiatric Association does not consider HTPs an addiction or a mental disorder at this time [37], it is of utmost importance to respond to the need for more research on this issue by developing a HTPs addiction scale based on criteria for Tobacco Use Disorder (TUD) found in the DSM-5 [37]. In the Diagnostic and Statistical Manual of Mental Disorders, fifth edition (DSM-5), the TUD diagnosis is assigned to individuals who are dependent on the drug nicotine due to use of tobacco products. By analogy, we propose to draw inspiration from these criteria to define and measure HTPs use.

### Aim of the present study

This study was motivated by the current lack of measures to evaluate HTPs addiction, and the obvious need to create one. This would contribute to combat misleading and misinterpreted findings from tobacco industry-drive studies, which have mainly employed measurement instruments suffering from design flaws [14, 38]. The main objectives were the following: (1) to design a questionnaire for HTPs addiction called “*Hea*ted *T*obacco *P*roducts *A*ddiction *Q*uestionnaire (HeaTPAQ)”, (2) to examine the psychometric properties of the newly developed HTPs addiction scale in terms of factor structure, internal consistency reliability, measurement invariance, convergent and concurrent validity. The study hypotheses are that: (a) using exploratory and confirmative factorial analysis techniques, the HeaTPAQ will yield a unidimensional factor structure, consistently with previous measures of nicotine addiction (e.g., the FTND [34], the CDS [33]); (b) the questionnaire will show good reliability estimates (McDonald’s omega and Cronbach’s alpha values exceeding 0.7 [39]); (c) the HeaTPAQ will demonstrate good convergent validity against another measurement of nicotine addiction (i.e., the FTND), and adequate concurrent validity with measures of depression and anxiety based on empirical evidence and theoretical considerations [40].

## Methods

Ethics approval for this study was obtained from the ethics committee of the School of Pharmacy at the Lebanese International University. Inclusion criteria were the following: being an adult from the general population aged 18 or older, originating from and residing in Lebanon at the time of the study, being able to read Arabic, currently reporting the use of a HTP (the IQOS brand), having access to the Internet, and providing an informed consent. Excluded were those who did not meet inclusion criteria, who did not consent to take part in the study, or who failed to complete the entire survey. A descriptive, observational and cross-sectional study was carried out during the period from May to July 2024. The snowball sampling technique was adopted to collect data via a Google Form link. Potential eligible participants, based on the before-mentioned inclusion criteria, were contacted by the research team via social apps (WhatsApp, Messenger, Instagram), inviting them to take part in the study. The contact details were obtained from the list of phone numbers of each member of the research team. The initial participants who agreed to participate were asked to forward the link to other participants. This process allowed a bigger sample, as each participant could recruit additional individuals. An introductory paragraph was included at the beginning of the link explaining the study’s objectives and containing a digital informed consent. Participants were assured about anonymity and confidentiality of their responses, and were asked to complete the survey voluntarily without compensation. The study questionnaire contained the following information and measures:

### Sociodemographic information

The questionnaire collected sociodemographic data consisting of age, gender, marital status, education level, cigarette smoking, IQOS smoking, in addition to the number of IQOS smoked per day and the years of IQOS smoking.

### The Fagerström test for nicotine dependence (FTND)

The FTND is composed of six items, three multiple-choice measured from 0 to 3 and three dichotomous (yes/ no) scored 0 and 1. Higher total scores indicate more intense dependence on nicotine [41]. The FTND was used in its Arabic validated version [42], and had a Cronbach’s alpha of 0.69 in the present study.

### The generalized anxiety disorder 7-item (GAD-7)

This is a self-report scale composed of 7 items (e.g., “Worrying too much about different things”) intended to measure the severity of generalized anxiety symptoms over the last two weeks according to the DSM-5 [43]. Items are scored from 0 (Not at all) to 3 (Nearly every day). Total scores range from 0 to 21. The Arabic validated version was adopted in the present study [44, 45], and yielded a Cronbach’s alpha of 0.92.

### The patient health Questionnaire-9 (PHQ-9)

The PHQ-9 was used to assess the severity of depression symptoms depression over the last two weeks through 9 items (e.g., “Feeling down, depressed, or hopeless”) [46]. Items are rated on 4 points from 0 (not at all) to 3 (nearly every day). Total scores vary from 0 to 27. Higher scores reflect more severe depression. The Arabic validated version was used [44, 47], with a Cronbach’s alpha of 0.92.

### The caffeine use disorder questionnaire (CUDQ)

The Arabic validation of the CUDQ was used (α = 0.90) [48]. This measure contains 10 items that were developed based on the DSM-5 criteria proposed for caffeine use disorder [49]. Items evaluate caffeine addiction symptoms experienced over the past year via a score varying from 1 (Never) to 4 (Very often).

#### Items construction and face validity

The construction of the HeaTPAQ was done following several steps. First, relevant literature was extensively reviewed to examine measurement tools available to assess nicotine addiction. Then, an initial pool of 24 items was constructed (Appendix). Six items were adapted from the FTND (items 2, 3, 18, 20, 22 and 24) [41], where any reference to smoking and nicotine was replaced with HTPs. Another 18 items were self-developed by the authors based on the DSM-5 criteria for TUD [37], with the aim of precisely assessing the main features of nicotine addiction outlined in the DSM-5 and not covered by the FTND: “Craving, or strong desire, to use HTP” (items 1 and 4), “physical dependence, or withdrawal symptoms” (items 10, 11, 12), “reduced control over HTP use” (items 17, 21 and 23), “tolerance development” (items 5 6, 7, 8, 9), “risky use” (item 19), and “social problems” (items 13, 14, 15, 16). The item pool was ensured to be a rich source that is relevant to the content of interest. Other important considerations were taken into account when developing items, including the avoidance of lengthy, double-barreled, or negatively worded items.

In accordance with TUD criteria [37], respondents were instructed to indicate whether they have experienced each of the HTP-related problems over the past year. A Likert format has been decided for scaling of items, with five response options: Strongly disagree (1), Disagree (2), Neutral (3), Agree (4) and strongly agree (5). The questionnaire heading specified that “HTPs” referred to a product that heats tobacco to produce an aerosol that can be inhaled. Besides, IQOS was given as reference, as it was introduced in Lebanon in 2022, and it represents the only HTP brand currently available in the country. The questionnaire was developed and administered to participants in the Arabic (native) language.

An expert panel comprising five experts on clinical psychology and psychiatry assessed all items for conciseness and clarity. Each expert was provided with pool of items initially obtained and asked to assess the relevance and comprehensiveness of each item using a rating scale from 1 (not relevant) to 5 (highly relevant), while also encouraged to provide qualitative feedback for any clarifications or revisions needed. After the expert review, the preliminary scale was sent out to 30 young adults, who were asked to report their perceptions of the scale’s applicability, understandability, and relevance.

#### Analytic strategy

There were no missing responses in the dataset. We used the exploratory-confirmatory (EFA-CFA) factor analyses technique to examine the factor structure of the scale (Swami & Barron, 2019). We split the main sample using the random option in SPSS into two subsamples; subsample 1 consisting of 33% of the participants used for the EFA (*n* = 246; mean age 27.82 ± 9.38 years) and subsample 2 consisting of 67% of the participants used for the CFA (*n* = 508; mean age 27.81 ± 8.80 years). There were no significant differences between the two subsamples in terms of mean age, *t*(752) = − 0.02, *p* =.982 and gender χ²(1) = 2.35, *p* =.125.

##### Exploratory factor analysis on the first subsample

The KMO and Bartlett’s statistics were assessed to check the suitability of the data. The Measure of Sampling Adequacy (MSA) at the item level was used to check whether an item needs to be eliminated from the analysis if values were below 0.50 [50]. The residual correlation between two items (referred to as doublets) was assessed via the Expected Residual correlation direct Change (EREC) index, which should be approximately 0. Items that repeatedly appear in different doublets were to be removed [51]. EFA was carried out with a polychoric correlation matrix given the ordinal nature of the variables and the high number of items with kurtosis and skewness values greater than|1 [52]. The method of estimation was Unweighted Least Squares (ULS), as recommended by international guidelines [53]. The Parallel Analysis (PA) was used to assess the number of factors to be retained [54, 55]. Loading factors ≥ 0.4 were considered adequate [56].

##### Confirmatory factor analysis on the second subsample

CFA was conducted via SPSS AMOS v.29 software. The minimum sample size for the CFA was esteemed at 72–480 participants based on 3 to 20 times the number of the scale’s variables [57]. We intended to test the factor structure we obtained in the EFA. Parameter estimates were obtained using the maximum likelihood method. The model adequacy was verified via several fit indices: the root mean square error of approximation (RMSEA) (≤ 0.08), standardized root mean square residual (SRMR) (≤ 0.05), the Tucker-Lewis Index (TLI) and the comparative fit index (CFI) (both ≥ 0.90) [58]. Multivariate normality was not verified (Bollen-Stine bootstrap *p* =.002); therefore, we performed non-parametric bootstrapping procedure.

##### Gender invariance

To examine gender invariance of HeaTPAQ scores, we conducted multi-group CFA [59] using the total sample. Measurement invariance was assessed at the configural, metric, and scalar levels [60]. We accepted ΔCFI ≤ 0.010 and ΔRMSEA ≤ 0.015 or ΔSRMR ≤ 0.010 as evidence of invariance [61].

The remaining analysis was done via SPSS software v.26. Composite reliability was assessed using McDonald’s ω and Cronbach’s α, with values greater than 0.70 reflecting adequate reliability. Normality of the HeaTPAQ total score was verified since the skewness ( = − 0.058) and kurtosis ( = − 0.544) values varied between − 1 and + 1 [62]. Consequently, the Pearson test was used to correlate two continuous variables and the independent sample t test to compare the HeaTPAQ total scores between sexes. *P* <.05 was deemed statistically significant.

## Results

### Characteristics of the sample

The total sample included 754 participants, with a mean age of 27.81 ± 8.99 years [min = 18; max = 62], a mean HCI of 1.04 ± 0.93 person/room, 52.8% males, 73.7% single and 80.5% with a university level of education (Table 1). All participants were current dual users of conventional cigarettes and IQOS, with a mean duration of IQOS use of 3.44 years. The mean HeaTPAQ score was 23.34 ± 11.88, with a median of 24, a minimum of 0 and a maximum of 52.

**Table 1:** Characteristics of the sample of IQOS smokers (*n* = 754)

Age categories^¥^	
Young adults (18–24 years)	360 (47.7%)
Early adulthood (25–34 years)	253 (33.6%)
Mid adulthood (35–44 years)	75 (9.9%)
Midlife (45–54 years)	53 (7.0%)
Late middle age (55–64 years)	13 (1.7%)
**Sex**	
Male	398 (52.8%)
Female	356 (47.2%)
**Marital status**	
Single	556 (73.7%)
Married	198 (26.3%)
**Education**	
Secondary or less	147 (19.5%)
University	607 (80.5%)
Exclusive cigarette smokers (yes)	212 (28.1%)
Dual smokers (IQOS + cigarette)	212 (100%)
Number of IQOS smoked per day	5.12 ± 5.79
Number of years of IQOS smoking	3.44 ± 3.32
Age (years)	27.81 ± 8.99 years [min = 18; max = 62]
Household crowding index (person/room)	1.11 ± 2.02
HeaTPAQ scores	23.34 ± 11.88

HeaTPAQ: Heated Tobacco Products Addiction Questionnaire. ¥ Age categories divided according to: [63].

### Exploratory factor analysis (subsample 1)

None of the items was suggested to be removed because of low MSA. However, the doublets identified through the EREC index led to the removal of items 1, 3, 4, 5, 6, 7, 9, 16, 21, 22 and 24 as they appeared the most frequently in the doublets. Another factor analysis was then conducted with the final 13 items. The KMO index (KMO = 0.969) and Bartlett’s test (*p* ≤.001) confirmed the adequacy of the data for the factor analysis. The parallel analysis indicated an adequate fit to one factor, which explained variance of 68.70%. Results indicated an adequate fit to a unidimensional structure supported by the GFI (GFI = 0.999) and CFI (= 0.999) being greater than 0.95, the UniCo (UniCo = 0.998) indice greater than 0.95, the I-ECV (I-ECV = 0.962) greater than 0.85 and MIREAL (MIREAL = 0.131) lower than 0.30.

### Confirmatory factor analysis (subsample 2)

CFA results showed that the unidimensional structure of the scale was very good: RMSEA = 0.079 (90% CI 0.071, 0.086), SRMR = 0.029, CFI = 0.962 and TLI = 0.955. The loading factors resulting from the EFA and CFA are summarized in Table 2. The Average Extracted Variance (AVE) value was 0.55 (> 0.5), indicating convergent validity of the scale.

The composite reliability was excellent in both subsamples (Table 2) and in the total sample (ω = 0.96 / α = 0.96).

**Table 2:** Loading factors of the heatpaq scale deriving from the exploratory factor analysis (EFA) and standardized loading factors deriving from the confirmatory factor analysis (CFA)

	EFA	CFA
1. I would have trouble getting the day started without IQOS.	0.77	0.74
2. I have gradually increased the amount of IQOS use from the first time I started using it.	0.69	0.72
3. Without my usual dose of IQOS, I would feel sick.	0.85	0.84
4. If I do not use IQOS, I would feel discomfort, sadness, difficulty sleeping and concentrating.	0.88	0.84
5. If I abstain from IQOS, I would become irritable and restless.	0.83	0.81
6. I use IQOS when I have to perform an important task.	0.81	0.80
7. I often fail to do things that I am supposed to do due to IQOS use.	0.86	0.84
8. I would not be able to function without using IQOS.	0.84	0.83
9. I could not stop using IQOS despite having troubles with family or friends.	0.82	0.81
10. I have difficulty refraining from using IQOS in places where its use is not allowed, such as in the library.	0.85	0.83
11. I would continue using IQOS even if I develop related health problems.	0.81	0.82
12. I use IQOS even when I am ill (like flu, colds, etc.) and have to stay in bed most of the day.	0.78	0.79
13. I have already tried to quit IQOS, but failed.	0.78	0.73
Cronbach’s α	0.96	0.96
McDonald’s ω	0.96	0.96

### Sex invariance

We were able to show the invariance across sex at the configural, metric, and scalar levels (Table 3). No significant difference was found between males and females in terms of HeaTPAQ scores (23.41 ± 11.86 vs. 23.26 ± 11.91; t(752) = 0.17; *p* =.864).

**Table 3:** Measurement invariance of the heatpaq scale across sex in the total sample

Model	CFI	RMSEA	SRMR	Model Comparison	ΔCFI	ΔRMSEA	ΔSRMR
Configural	0.957	0.059	0.034				
Metric	0.958	0.056	0.034	Configural vs. metric	0.001	0.003	< 0.001
Scalar	0.958	0.054	0.034	Metric vs. scalar	< 0.001	0.002	< 0.001

Note. CFI = Comparative fit index; RMSEA = root mean square error of approximation; SRMR = Standardised root mean square residual

### Concurrent validity

Higher total HeaTPAQ scores were significantly associated with higher cigarette dependence (FTND scores; *p* <.001), caffeine addiction (*p* <.001), depression (*p* <.001) and anxiety (*p* <.001) (Table 4).

No significant difference was found between age categories in terms of IQOS dependence: young adults (18–24 years; 22.67 ± 12.25), early adulthood (25–34 years; 23.50 ± 11.26), mid adulthood (35–44 years; 24.59 ± 12.17) midlife (45–54 years; 25.70 ± 11.56) and late middle age (55–64 years; 22.08 ± 12.48), F(4,749) = 1.07, *p* =.372, Cohen’s d = 0.006.

**Table 4:** Pearson correlation matrix between continuous variables

	1.	2.	3.	4.
1. HeaTPAQ scores	1			
2. FTND scores	0.24***	1		
3. CUDQ scores	0.22***	0.20**	1	
4. Anxiety	0.26***	0.27***	0.39***	1
5. Depression	0.27***	0.22**	0.41***	0.75***

HeaTPAQ: Heated Tobacco Products Addiction Questionnaire; FTND: Fagerström test for nicotine dependence; CUDQ: Caffeine Use Disorder Questionnaire; **p <.01; ***p <.001

## Discussion

Public health experts currently agree that HTPs pose a significant health risk for their consumers because they contain carcinogens, heavy metals, and nicotine [12, 64]. The same concentrations [23, 24, 65] and speed of delivery [25] of nicotine found for HTPs and conventional combustion cigarettes make it necessary to consider the addictiveness of HTPs, and provide precise diagnostic instruments to serve as the basis for effective treatment plans. This study’s goal was to construct and validate a novel measure to define and assess patterns of addictive HTPs use by adapting TUD DSM-5 criteria. As anticipated, findings showed that the HeaTPAQ has a single-factor structure with 13 items, an excellent internal consistency, as well as good convergent and concurrent validity. Thus, our preliminarily validation study suggests that the HeaTPAQ is useful and suitable to evaluate HTPs addiction in general population adults.

Exploratory, then confirmatory factor analyses were applied and revealed a one-factor model consisting of 13 items with acceptable fit to the data. Additionally, the HeaTPAQ reached excellent internal consistency coefficients, with both Cronbach’s α and McDonald’s ω values of 0.96. This provides empirical support to the hypothesized unidimensional structure of the scale, with all items together assessing the same underlying construct of HTPs addiction and in the same direction. This result is compatible with previous instruments designed to measure nicotine addiction and found to be unidimensional in structure, such as the CDS [33] or the Smoking Scale [66]. The findings also align with the DSM criteria for tobacco dependence, which require that any two of 11 problematic patterns of tobacco use must be present to identify an individual with TUD [37]. This implies that the constellation of symptoms identified by the DSM are expected to be associated. Overall, unidimensionality suggests that scores of all 13 items should be summed together to generate a single meaningful HeaTPAQ total score [67], and guarantees that the measurement made about the overall HTPs addiction under consideration is sound [68].

The one-dimensional structure of the HeaTPAQ was found to be invariant across sex groups, which means that the scale functioned equally for males and females and its items were interpreted in a conceptually similar way by both sexes. Establishing this psychometric property allows ensuring that the HeaTPAQ is a precise and accurate measure for use in future research to make between-groups comparisons of HTPs addiction. No statistically significant differences were found between male and female participants in HTPs addiction. Broadly in line with our results, a large multinational study performed in 28,300 individuals aged over 15 years in 28 European countries found that being male was significantly linked to increased odds of ever HTP consumption, but no significant sex differences were observed in current and daily HTP consumption [69].

Convergent validity of the HeaTPAQ was demonstrated through significant positive correlation with FTND scores, thus suggesting that both scales tend to measure similar constructs (i.e., addiction to nicotine contained in heated tobacco versus regular cigarettes). It is of note that the total sample reported dual-use of IQOS and conventional cigarettes, and have been IQOS consumers since a mean duration of 3.44 years. This finding is consistent with data from other parts of the world documenting a high risk of dual use of IQOS and conventional cigarettes among smokers who attempt to quit [13, 14]. This is not a negligible problem, since dual users of cigarettes and HTPs were found to be less likely to quit tobacco relative to exclusive cigarette smokers [70]. Furthermore, HeaTPAQ scores correlated positively and as expected with measures of anxiety and depression, which suggests the adequate concurrent validity of the scale. In fact, prior research has shown that there is a well-established connection between nicotine addiction and depression/anxiety [71, 72]. The use of nicotine may lead to development of depression and anxiety symptoms through cholinergic hypersensitivity, or a hyperactivation of cholinergic signaling [73]. These findings build on, and extend previous evidence on the patterns of nicotine addiction and HTPs use in relation to psychopathology [40].

Finally, higher HeaTPAQ scores were associated with higher caffeine addiction scores in our sample. This is in line with previous evidence suggesting that caffeine consumption is significantly linked to a greater likelihood of smoking craving in HTPs smokers [74]. Other previous research reported that caffeinated beverages enhanced smoking taste [75]. Some biochemical mechanisms have been proposed to explain why caffeine addiction is related to HTPs addiction. As an adenosine receptor antagonist, caffeine inhibits adenosine and stimulates dopamine release, thus affecting glutamate, gamma-aminobutyric acid and dopamine levels [76]. Therefore, and as an addictive substance, caffeine interacts with smoking in a way that caffeine metabolism is increased by smoking and that nicotinic acetylcholine receptors is affected by n-MP, a biomarker of coffee consumption [77–79].

### Study limitations

Despite its significant contribution to current literature, our study has some limitations that should be addressed in future research. Self-report measures were used to gather data from participants. Future studies should consider using clinical interviews to determine whether participants met the diagnostic criteria. Moreover, a web-based snowball sampling was adopted, which may limit the generalizability of our conclusions. Future studies need to be carried-out in larger and more representative samples of HTPs users from other countries and settings, while using longitudinal-experimental designs, and including other HTP brands. In addition, this study only accounted for traditional cigarettes, and no information was collected regarding other tobacco products (such as e-cigarettes or waterpipe). This point needs to be addressed in future research. Finally, other psychometric characteristics, such as test-retest reliability and predictive validity, still need to be tested in further studies.

### Practical implications and future perspectives

Despite their documented harmful effects on health and their high addictive potential, the promotion and advertising of HTPs via the Internet and social media are not banned in many countries [80], which has led to their growing in popularity, especially among younger people. In Lebanon, for example, the prevalence of cigarette smoking (35.1%) is among the highest worldwide [81]. The heated tobacco market remains uncontrolled and unregulated in any way in the country, especially in the current economic crisis that Lebanon is going through, and which has deprioritized tobacco control [82]. In the face of this under-recognized and uncontrolled scourge, many clinicians seem unprepared to provide consistent and informed advice to patients and families about HTPs use [32]. Offering the HeatPAQ as a valid and reliable measure for routine use in clinical and research practice can drive awareness and engagement of clinicians, researchers and policy-makers in public debate and action about HTPs-related health risks. The implementation of routine screening of HTPs addiction using the new scale in smoking cessation services, in combination with educational interventions, can help change the public’s perception of HTPs as safe and harmless. Healthcare providers should understand, help inform and raise awareness on health and dependence risks related to HTPs use, and encourage the adoption of more effective and safer treatments to help quit smoking (nicotine replacement therapy, varenicline, bupropion, combined with psychological counselling) [31]. Finally, using the HeatPAQ in future research can enrich the evidence to negate the widespread misperception that HTPs are effective as a smoking cessation aid, and the misperception of HTPs users who tend to not view themselves as tobacco users [83]. This would strengthen public health efforts toward denormalization of the usage of all types of tobacco and reliance instead on evidence-based cessation resources [84]. Therefore, the scale needs to be translated, culturally adapted and validated in other languages to further confirm its structural characteristics and psychometric properties, and so that clinicians and researchers may benefit from using the HeatPAQ to measure HTPs addiction in young adult consumers throughout the world.

## Conclusion

As independent scientists, we attempted through this study to contribute to the collective efforts aimed at enlightening the public on addiction risks and health harms of HTPs, by creating and preliminarily validating - for the first time - a new measure of HTPs addiction. Findings suggest that the HeatPAQ is a specific, short and simple-to-use self-report questionnaire to assess HTPs addiction reliably and validly. Pending future studies confirming our results, we hope that the HeatPAQ will facilitate routine screening for HTPs addiction, which is an essential step towards appropriate prevention and intervention efforts and to inform policy makers. We also hope that the HeatPAQ will help drive awareness and educate young people on the potential health risks of high consumption of HTPs.

## Electronic supplementary material

Below is the link to the electronic supplementary material.

Supplementary Material 1

## Data Availability

All data generated or analyzed during this study are not publicly available due to restrictions from the ethics committee, but are available upon a reasonable request from the corresponding author (SH).
