# Supplementary material for: Is it about substituting an addiction with another? development and initial psychometric properties of the first heated tobacco products addiction questionnaire (HeaTPAQ)
Source: Addict Sci Clin Pract. 2025 Feb 26;20:20. doi: 10.1186/s13722-025-00551-4 (PMC11863451; doi:10.1186/s13722-025-00551-4)
Supplement: Supplementary file 1 — Supplementary Material 1 [file 13722_2025_551_MOESM1_ESM.docx]

**Appendix.** English items of the Heated Tobacco Products Addiction Questionnaire (HeaTPAQ)

|  | **5 = Strongly agree** | **4 = Agree** | **3 = Neutral** | **2 = Disagree** | **1 = Strongly disagree** |
| --- | --- | --- | --- | --- | --- |
| 1. I frequently crave IQOS. **^b*^** |  |  |  |  |  |
| 1. I would have trouble getting the day started without IQOS. **^a^** |  |  |  |  |  |
| 1. I generally start using IQOS during the first hour after waking up. **^a*^** |  |  |  |  |  |
| 1. I have a strong desire for IQOS. **^b*^** |  |  |  |  |  |
| 1. I could hardly get through the day without IQOS. **^b*^** |  |  |  |  |  |
| 1. I need IQOS every day. **^b*^** |  |  |  |  |  |
| 1. I find myself using IQOS more and more frequently over time. **^b*^** |  |  |  |  |  |
| 1. I have gradually increased the amount of IQOS use from the first time I started using it. **^b^** |  |  |  |  |  |
| 1. I have ended up using IQOS in situations and times when I did not plan to. **^b*^** |  |  |  |  |  |
| 1. Without my usual dose of IQOS, I would feel sick. **^b^** |  |  |  |  |  |
| 1. If I do not use IQOS, I would feel discomfort, sadness, difficulty sleeping and concentrating. **^b^** |  |  |  |  |  |
| 1. If I abstain from IQOS, I would become irritable and restless. **^b^** |  |  |  |  |  |
| 1. I use IQOS when I have to perform an important task. **^b^** |  |  |  |  |  |
| 1. I often fail to do things that I am supposed to do due to IQOS use. **^b^** |  |  |  |  |  |
| 1. I would not be able to function without using IQOS. **^b^** |  |  |  |  |  |
| 1. When I have to choose, I would prefer using IQOS over leisure activities or social events. **^b*^** |  |  |  |  |  |
| 1. I could not stop using IQOS despite having troubles with family or friends. **^b^** |  |  |  |  |  |
| 1. I have difficulty refraining from using IQOS in places where its use is not allowed, such as in the library. **^a^** |  |  |  |  |  |
| 1. I would continue using IQOS even if I develop related health problems. **^b^** |  |  |  |  |  |
| 1. I use IQOS even when I am ill (like flu, colds, etc.) and have to stay in bed most of the day. **^a^** |  |  |  |  |  |
| 1. It would be difficult for me to limit how much I use IQOS. **^b*^** |  |  |  |  |  |
| 1. I find it difficult to abstain from IQOS use. **^a*^** |  |  |  |  |  |
| 1. I have already tried to quit IQOS, but failed. **^b^** |  |  |  |  |  |
| 1. I cannot imagine myself completely quitting IQOS. **^a*^** |  |  |  |  |  |

**^a^** Fagerström.

**^b^** Self-developed.

*Items removed.
